# Microsatellite‐Based Genetic Analysis of a Wild Alpine Bumblebee and the Environmental Drivers of Its Genetic Diversity

**DOI:** 10.1002/ece3.72876

**Published:** 2026-01-05

**Authors:** Rui Zhang, Guo Sun, Chengbo Liang, Daoxin Liu, Jingyan Yan

**Affiliations:** ^1^ College of Eco–Environmental Engineering Qinghai University Xining China; ^2^ Sichuan Lomonbio Technology Co. Ltd., Sichuan Engineering Technology Research Center of Biological Pesticide Meishan Sichuan China; ^3^ Qinghai Provincial Key Laboratory of Animal Ecological Genomics Xining China; ^4^ College of Agriculture and Animal Husbandry Qinghai University Xining China

**Keywords:** bumblebees, environmental association, genetic diversity, microsatellite markers, population structure

## Abstract

*Bombus kashmirensis* is a key ecological indicator species on the Qinghai–Tibet Plateau, and clarifying its genetic characteristics in Qinghai Province is essential for supporting bumblebee conservation and management efforts in this region. In this study, we analyzed the genetic diversity, population structure, and environmental factors influencing genetic diversity in 539 *B. kashmirensis* individuals from 36 geographic populations using 15 SSR markers. A total of 382 alleles were identified across the 15 SSR loci, with an average polymorphic information content (*PIC*) of 0.85, indicating a high level of genetic polymorphism. The populations also exhibited high genetic diversity, with mean values of the number of alleles (*N* *a*) = 10.03, Shannon's information index (*I*) = 1.98, and unbiased expected heterozygosity (*uHe*) = 0.84. Genetic differences among populations were generally low but variable, with Nei's genetic distance ranging from 0.130 to 0.710 and *F*st values between 0.013 and 0.078. Consistent results from STRUCTURE, UPGMA, and PCoA analyses revealed two major genetic clusters corresponding to eastern and western regions. AMOVA at *K* = 2 further supported this pattern, showing that genetic variation between groups (2.00%) exceeded that among populations within groups (1.42%). Pearson correlation analysis identified longitude, latitude, solar radiation (July and September), and water vapor pressure (September) as significant environmental factors shaping population genetic diversity. These results provide essential baseline data for understanding the genetic diversity of *B. kashmirensis* and offer a scientific basis for its regional conservation and management.

## Graphical abstract

High genetic diversity was detected in 539 *B. kashmirensis* individuals from 36 populations in Qinghai Province using 15 SSR markers. STRUCTURE, UPGMA, and PCoA analyses revealed east–west genetic clustering of populations. Environmental factors such as latitude, longitude, solar radiation, and vapor pressure influence genetic diversity.

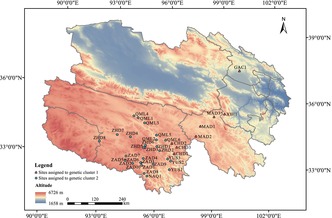

## 1 Introduction

Genetic variation is the foundation of biological evolution and plays a critical role in species' adaptation potential to environmental changes and the preservation of population stability (Arber 2000). As essential components of ecosystems, pollinating insects contribute indispensably to the stability of plant communities and the facilitation of plant reproduction (Li et al. 2022). The extent of genetic variation within a population is a key determinant of its ability to adapt to changing environments (Lande and Shannon 1996). The genetic diversity and population structure of pollinating insects are shaped not only by geographical factors but also by host plant isolation, historical distribution processes, and external environmental selection pressures (Hu et al. 2023). These factors interact to collectively shape the genetic landscape of populations. In recent years, global declines in pollinating insect abundance have been driven by habitat loss and fragmentation, widespread pesticide use, pathogen transmission, and climate change (Kerr et al. 2015). This trend has indirectly led to a reduction in their genetic diversity, thereby weakening the adaptive capacity of populations to environmental changes (Espregueira Themudo et al. 2020; Le Conte and Navajas 2008). Therefore, enhancing the conservation of pollinating insects' genetic diversity is of paramount importance for sustaining ecosystem services.

Bumblebees (*Bombus* spp.) belong to the phylum Arthropoda, class Insecta, order Hymenoptera, family Apidae, and genus *Bombus*, comprising 15 recognized subgenera (Williams et al. 2008). As one of the key pollinator groups for numerous wild plants and crops, bumblebees play vital roles in both natural ecosystems and agricultural production (An et al. 2010). The transition zone from the eastern Qinghai–Tibet Plateau to the Loess Plateau, Qinling Mountains, and Sichuan Basin is one of the global hotspots of bumblebee diversity (An et al. 2011). Qinghai Province, located in the northeastern part of the Qinghai–Tibet Plateau, is an important region within this transitional zone, characterized by a cold climate and vegetation dominated by alpine meadows (Dai ZiJun et al. 2018). Its distinctive high‐altitude environment makes bumblebees one of the dominant pollinator groups. *B. kashmirensis* , belonging to the subgenus *Alpigenobombus* (Huang and An 2018), is widely distributed in regions such as Maduo County, Zhidoi County, and Yushu City in Qinghai Province (Cheng‐bo et al. 2022). It serves as a key pollinator in this area and also as an important ecological indicator species that reflects the environmental conditions and ecosystem health of the Qinghai–Tibet Plateau (Naeem et al. 2020).

Simple sequence repeat (SSR) markers are tandemly repeated DNA sequences that are widely distributed throughout eukaryotic genomes. Owing to their high polymorphism, codominant inheritance, broad genome coverage, and ease of experimental application, SSR markers have been extensively employed in studies of genetic diversity, population structure, and kinship analysis (Vieira et al. 2016; Senan et al. 2014; Abdul‐Muneer 2014; Tang et al. 2007; Menezes et al. 2020; Li et al. 2005). Previous studies have shown that SSR markers provide high resolution in genetic analyses of bumblebee populations and remain among the most commonly used and effective molecular tools to date. For instance, Widmer et al. (1998) analyzed bumblebee populations in north‐central Europe using six SSR loci, revealing high levels of genetic variation and heterozygosity, along with significant genetic differentiation driven by the geographic barrier of the Alps, which led to the formation of two distinct gene pools; Estoup et al. (1996) analyzed *B. terrestris* populations across Europe and nearby islands using microsatellite and mitochondrial markers, revealing high genetic polymorphism and significant differentiation between continental and island populations, which were shaped by island isolation, historical bottlenecks, and restricted gene flow. These previous studies based on SSR markers not only demonstrate the reliability of this method in resolving population genetic structure but also underscore the pivotal roles of geographical barriers and ecological factors in shaping genetic patterns. Environmental factors such as altitude (Hodkinson 2005) and temperature (Sinclair et al. 2012) have been shown to influence population genetic structure and gene flow. Therefore, the combined effects of these environmental factors should be carefully considered in assessments of genetic diversity.

In this study, we conducted a comprehensive analysis of the genetic diversity and population structure of *B. kashmirensis* across multiple geographic populations in Qinghai Province using SSR molecular markers. By integrating environmental and geographical variables, we further evaluated the key factors shaping the genetic diversity of the species. This study aims to elucidate the patterns of genetic variation in bumblebee populations inhabiting high‐altitude regions and to clarify their associations with environmental factors. The findings are expected to provide a scientific basis and data support for the long‐term genetic monitoring, conservation, and management of *B. kashmirensis* .

## 2 Materials and Methods

### 2.1 Sample Collection and DNA Extraction

**FIGURE 1:**
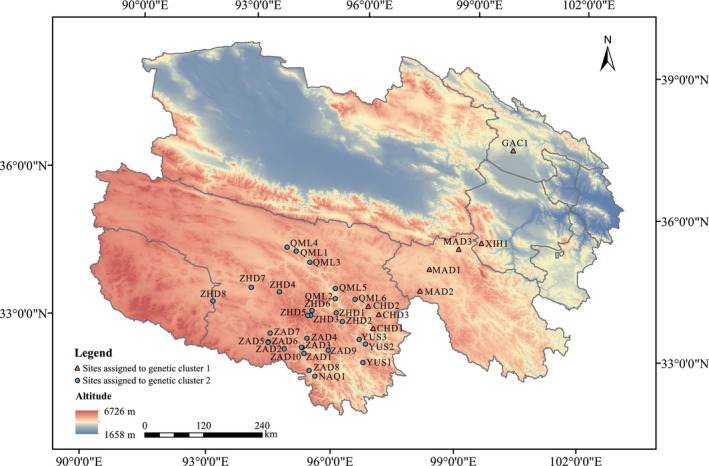
Geographic locations of the *B. kashmirensis* sampling sites. Sites assigned to genetic cluster 1 are shown as red triangles, and sites assigned to genetic cluster 2 are shown as blue circles.

Between July and August 2022, *B. kashmirensis* worker specimens were extensively collected across Qinghai Province using aerial netting. At each sampling site, individuals were collected within a 1 km^2^ area over a standardized 1h period. Location coordinates and site‐specific information were recorded and labeled accordingly (Figure 1). A total of 539 individuals were collected from 36 sampling sites, with 7 to 24 individuals captured per site. All specimens were immediately preserved in 90% ethanol in the field and subsequently transported to the laboratory for further analyses. Species identification was based on morphological characteristics and further confirmed by mitochondrial COI DNA barcoding, ensuring that all samples belonged to *B. kashmirensis* . Genomic DNA was extracted from thoracic muscle tissue using the *Ezup Column Animal Genomic DNA Extraction Kit* (Sangon Biotech Co. Ltd., Shanghai, China) according to the manufacturer's protocol. DNA quality was assessed using a microvolume spectrophotometer and 1% agarose gel electrophoresis. Samples showing a clear, high‐molecular‐weight genomic DNA band with no visible degradation or nonspecific bands were considered high quality and stored at −20°C for downstream applications.

### 2.2 SSR Primer Screening and PCR Amplification

In our previous study (Guo et al. 2024), we used MISA software (Beier et al. 2017) to analyze the unigene sequences of the *B. kashmirensis* transcriptome to identify potential SSR loci. The parameters were set as follows: the minimum number of repeat units was 10 for mononucleotide repeats, 6 for dinucleotide repeats, and 5 for tri‐, tetra‐, penta‐, and hexanucleotide repeats. A total of 27,414 SSR loci were identified, with mono‐, di‐, and trinucleotide repeats being the most abundant types. The identified SSR loci were subsequently imported into the Primer3Plus online tool (Untergasser et al. 2007) (https://www.primer3plus.com/) for SSR primer design. Primer design was performed with the following parameters: primer length 18–24 bp (maximum 27 bp), GC content 40%–60% (maximum 70%), melting temperature (*T*_m_) 50°C–61°C (maximum 63°C), primer bound % −10.0 to 97.0 (maximum 110.0), and PCR product size ranges of 101–200 bp, 201–300 bp, and 301–400 bp. To ensure diversity among primers, 30 primer pairs were randomly selected from those chosen SSR loci. These 30 primer pairs covered fragment length ranges of < 150 bp, 150–200 bp, and 200–300 bp, and represented loci with repeat numbers of 15–20, 21–25, 26–30, 31–35, and > 36. In addition, based on previously published literature (Karslı and Gürel 2020; Taye et al. 2020), seven microsatellite primer pairs suitable for *Bombus* were further selected. All primers were synthesized by Sangon Biotech Co. Ltd.

Ten worker bees of *B. kashmirensis* were used to validate the primers. Genomic DNA was extracted using the Ezup Column Animal Genomic DNA Extraction Kit (Sangon Biotech Co. Ltd.). PCR amplification was carried out in a 25 μL reaction mixture containing 0.5 μL genomic DNA template, 0.5 μL each of forward and reverse primers (10 μM), 12.5 μL of 2× Taq Master Mix, and 11 μL of ddH_2_O. The thermal cycling program consisted of an initial denaturation at 94°C for 1 min 30 s, followed by 35 cycles of 94°C for 20 s, 59°C for 20 s, and 72°C for 20 s, with a final extension at 72°C for 5 min. PCR products were visualized by electrophoresis on a 1.2% agarose gel at 110 V for 35 min. Ultimately, 15 primer pairs demonstrating strong amplification performance were selected (Table 1). These primers were labeled with FAM or HEX fluorescent dyes for subsequent PCR amplification. Each SSR locus was amplified separately in singleplex PCR reactions. Each reaction system (25 μL) contained 0.5 μL of *B. kashmirensis* DNA template, 0.5 μL each of forward and reverse primers (0.2–1.0 μM), 12.5 μL of 2× Taq Master Mix*, and 11 μL of ddH_2_O. The PCR program was as follows: initial denaturation at 94°C for 1 min 30 s; 35 cycles of denaturation at 94°C for 20 s, annealing at primer‐specific temperatures (49°C–60°C) for 20 s, and extension at 72°C for 20 s; followed by a final extension at 72°C for 5 min and a hold at 4°C.

**TABLE 1:** Information of 15 SSR loci used for *B. kashmirensis* .

Locus	Repeat motif	Forward primer (5′‐3′)	Reverse primer (5′‐3′)	*T*_m_ (°C)	Size range (bp)	Fluorescent label	Source
BK1	AG	CCGTTCATTACCCGACCATA	CGACATCAGGAAGTTTCTTCG	60	279	FAM	This study
BK4	AG	TTCAAGTTTGCATGCTCCAG	CCTGGCCTTCCTCTCTTTCT	60	213	HEX	This study
BK5	GA	GACGCGATACGACCTCTCTC	CATGTCGGCATATCAGTTGG	60	225	FAM	This study
BK9	GA	GCGAACGATCCTAATGTGGT	CGACTCGCGAACAACAACTA	60	153	FAM	This study
BK10	GA	AAGGCAGAGCGAGAGAATGT	CGAGCAGCCATCAGAATACA	60	161	HEX	This study
BK11	GA	CATCGACGATATGAGAACGC	CCCGCTAAAAATTCCCTCTC	60	109	FAM	This study
BK21	AG	GCGCGAGAATGTACATCAGA	CGTACGCGATCTTCTTTTCC	60	197	HEX	This study
BK24	TCG	GCTATGGTTGCTGCTGTGAA	GCGTGATCCACAATGACATC	60	164	FAM	This study
BK27	TATG	CCTGTTCTTGAGTTGTACCGA	GCGTTATCTAAACATTCACTCGAT	58	213	HEX	This study
BK28	TGTA	TTGGGTATAGTGCCATGAAATG	ATTGTAAAATTTGGGTGCGG	60	259	FAM	This study
BK30	AAGA	AGTTCCCGATGCTTTCACAC	GCTCCTTTCTTCCCTCCTTG	60	264	FAM	This study
BT10	CT	TCTTGCTATCCACCACCCGC	GGACAGAAGCATAGACGCACCG	53	151–175	HEX	Karslı et al.,2020
BT26	TG	AGCGGGACCTGGTAAAAACG	CGATTCTCTTCGTGGTCAGTTCTCC	52	119–191	HEX	Karslı et al.,2020
BTERN01	AG	CGTGTTTAGGGTACTGGTGGTC	GGAGCAAGAGGGCTAGACAAAAG	49	96–124	HEX	Taye et al.,2020
BTERN02	CT	TTTCCACCCTTCACGCATACAC	GATTTTATCCTCCGACCGTTCC	52	152–162	FAM	Taye et al.,2020

### 2.3 Gel and Capillary Electrophoresis Analysis

PCR products were first assessed by 1.2% agarose gel electrophoresis (110 V, 35 min), and amplification products with clear and bright bands were selected for further analysis. Selected samples were submitted to Sangon Biotech Co. Ltd. for capillary electrophoresis to obtain genotyping data for the microsatellite markers. The resulting electropherograms were manually inspected and edited using GeneMarker software (Hulce et al. 2011). SSR alleles were stringently filtered based on repeat motifs, expected fragment sizes, and peak quality characteristics. Amplification products exhibiting significant deviations from expected sizes, weak fluorescence signals, or obvious background noise were excluded. A high‐quality genotyping matrix of SSR loci was ultimately constructed from the filtered data and employed for downstream genetic analyses.

## 3 Data Analysis

### 3.1 Population Genetic Diversity Analysis

The curated genotyping data were organized and summarized using Microsoft Excel 2019. Genetic parameters for each studied population and SSR locus, including the observed number of alleles (*N* *a*), effective number of alleles (*N* *e*), Shannon's information index (*I*), observed heterozygosity (*H* *o*), expected heterozygosity (*H* *e*), unbiased expected heterozygosity (*uHe*), and fixation index (*F*), were calculated using GenAlEx v6.501 software (Peakall and Smouse 2012). The polymorphic information content (*PIC*) of the SSR loci was calculated using PowerMarker v3.25 software (Liu and Muse 2005).

### 3.2 Population Genetic Structure Analysis

Population genetic differentiation coefficient (*F*st) and Nei's genetic distances were calculated using GenAlEx software to quantify the level of genetic differences and genetic relationships among populations. To infer the underlying population structure, Bayesian clustering analysis of *B. kashmirensis* populations was conducted in STRUCTURE v2.3.4 (Earl and vonHold 2012), with the number of assumed genetic clusters (*K*) ranging from 1 to 10. The burn‐in period was set to 50,000 iterations, followed by 550,000 Markov Chain Monte Carlo (MCMC) repetitions. Each *K* value was replicated 10 times to evaluate the consistency of clustering results. The most likely number of clusters (optimal *K*) was determined based on the Δ*K* method of Evanno et al. (2005). In addition, a UPGMA dendrogram based on Nei's genetic distances was constructed using MEGA 7.0 (Kumar et al. 2016) to illustrate hierarchical relationships among populations. Principal Coordinate Analysis (PCoA) was also performed in GenAlEx to visualize genetic relationships among geographic populations. Furthermore, Analysis of Molecular Variance (AMOVA) was performed using ARLEQUIN v3.5 (Excoffier and Lischer 2010), based on the optimal number of clusters inferred from STRUCTURE (*K* = 2), to assess the partitioning of genetic variation among population groups.

### 3.3 Correlation Analysis Between Genetic Diversity and Environmental Variables

Geographic and environmental variables for each sampling site were extracted using toolsets in ArcMap 10.8 (Esri 2020). Pearson correlation analysis was performed to assess the relationships between six genetic diversity indices (*N* *a*, *N* *e*, *I*, *H* *o*, *H* *e*, and *uHe*) and three geographic variables (longitude, latitude, and elevation), as well as 27 environmental factors. The environmental variables included annual precipitation, annual average temperature, annual net primary productivity (NPP), inter‐patch dissimilarity, herbaceous vegetation cover, topographic position index, aspect, and monthly averages of precipitation, temperature, solar radiation, vapor pressure, and wind speed from June to September (4 months × 5 climatic variables = 20 factors). The environmental datasets used in this study are all publicly available from open‐access databases. Climatic variables were obtained from the WorldClim global climate database (https://www.worldclim.org) as mean values averaged over the period 1970–2000. Annual NPP data were sourced from the University of Montana (ftp://ftp.ntsg.umt.edu), while land cover, habitat heterogeneity, and topographic variables were retrieved from the EarthEnv database (https://www.earthenv.org). All environmental datasets were accessed in December 2024 and resampled to a spatial resolution of approximately 1 km^2^. Pearson correlation results were visualized using the *ggplot2* v3.5.1 package in R v4.4.1.

## 4 Results

### 4.1 Genetic Diversity of *B. kashmirensis* Population

Among the 15 SSR markers used, a total of 382 alleles (*N* *a*) were detected, with loci BK4 and BK1 exhibiting the highest number of alleles (42 each), while loci BK24 and BK28 showed the lowest number (14 each). The average number of alleles per locus was 25.47. Shannon's information index (*I*) ranged from 1.24 to 2.52, with a mean of 1.98. Observed heterozygosity (*H* *o*) varied between 0.59 and 0.91, averaging 0.80, while expected heterozygosity (*H* *e*) ranged from 0.59 to 0.90, with a mean of 0.81. Unbiased expected heterozygosity (*uHe*) ranged from 0.62 to 0.94, averaging 0.84. Polymorphism information content (*PIC*) values ranged from 0.62 to 0.95, all exceeding 0.50, with an average of 0.85 (Table 2). Overall, these markers demonstrated high polymorphism, indicating their suitability for population genetic structure analyses.

**TABLE 2:** Genetic diversity statistics of 15 SSR Loci in *B. kashmirensis* populations.

Locus	*N* *a*	*I*	*H* *o*	*H* *e*	*uHe*	*PIC*
BK1	42	2.18	0.77	0.85	0.88	0.89
BK4	42	2.47	0.81	0.90	0.93	0.94
BK5	41	2.52	0.88	0.90	0.94	0.95
BK9	18	1.85	0.84	0.81	0.84	0.84
BK10	26	2.20	0.88	0.87	0.90	0.90
BK11	30	2.35	0.89	0.89	0.92	0.92
BK21	20	2.02	0.86	0.84	0.88	0.88
BK24	14	1.43	0.73	0.69	0.72	0.70
BK27	24	1.92	0.84	0.81	0.84	0.83
BK28	14	1.64	0.76	0.77	0.80	0.79
BK30	16	1.80	0.82	0.79	0.82	0.83
BT10	24	2.07	0.91	0.85	0.88	0.90
BT26	21	1.82	0.73	0.78	0.81	0.86
BTERN01	29	2.14	0.76	0.82	0.85	0.92
BTERN02	21	1.24	0.59	0.59	0.62	0.62
Average	25.47	1.98	0.80	0.81	0.84	0.85

Among the *B. kashmirensis* populations, the *N* *a* per population ranged from 6.53 to 13.27, while the *N* *e* varied between 5.02 and 8.28. The *I*, *H* *o*, *H* *e*, and *uHe* ranged from 1.63 to 2.23, from 0.60 to 0.90, from 0.74 to 0.86, and from 0.79 to 0.88. The fixation index (*F*) varied between −0.11 and 0.25. Population QML5 (located in the central part of the study area) exhibited the highest values of *N* *a*, *I*, and *H* *e*, while population MAD3 (located in the eastern part of the study area) showed the lowest *N* *a*, *N* *e*, *I*, and *H* *e* values (Table 3).

**TABLE 3:** Genetic diversity of 36 populations of *B. kashmirensis* .

Population	*N* *a*	*N* *e*	*I*	*H* *o*	*H* *e*	*uHe*	*F*
ZHD1	11.87	7.60	2.14	0.80	0.84	0.86	0.05
ZHD2	11.93	7.72	2.14	0.85	0.84	0.86	−0.02
ZHD3	7.07	5.02	1.68	0.75	0.76	0.80	0.02
ZHD4	10.40	5.72	1.92	0.77	0.80	0.82	0.04
ZHD5	9.27	6.36	1.94	0.76	0.80	0.84	0.04
ZHD6	10.60	6.66	2.04	0.82	0.83	0.85	0.02
ZHD7	10.60	7.48	2.09	0.83	0.84	0.88	0.02
ZHD8	10.60	7.09	2.07	0.88	0.83	0.86	−0.06
ZAD1	8.87	6.58	1.93	0.77	0.81	0.85	0.04
ZAD2	12.27	8.28	2.18	0.79	0.85	0.87	0.08
ZAD3	9.07	6.15	1.92	0.60	0.80	0.84	0.25
ZAD4	11.73	7.48	2.13	0.87	0.84	0.86	−0.04
ZAD5	11.20	7.81	2.14	0.81	0.84	0.87	0.05
ZAD6	8.40	5.50	1.79	0.84	0.77	0.80	−0.11
ZAD7	10.67	7.92	2.08	0.80	0.83	0.86	0.03
ZAD8	9.40	6.83	1.99	0.90	0.83	0.87	−0.09
ZAD9	10.47	6.46	2.02	0.90	0.83	0.85	−0.09
ZAD10	6.80	5.10	1.70	0.76	0.77	0.83	0.02
YUS1	9.87	7.01	1.98	0.84	0.80	0.84	−0.05
YUS2	9.53	6.55	2.01	0.88	0.83	0.87	−0.06
YUS3	9.13	6.60	1.94	0.86	0.81	0.85	−0.08
QML1	12.13	5.80	2.09	0.86	0.82	0.84	−0.05
QML2	9.53	7.14	1.93	0.80	0.81	0.83	0.01
QML3	10.27	6.26	1.99	0.70	0.82	0.84	0.14
QML4	10.07	6.30	2.04	0.83	0.83	0.86	0.01
QML5	13.27	6.94	2.23	0.82	0.86	0.87	0.04
QML6	6.60	8.14	1.68	0.84	0.76	0.82	−0.11
NAQ1	11.40	5.04	2.12	0.82	0.84	0.87	0.03
XIH1	9.73	7.44	1.87	0.77	0.78	0.80	0.01
MAD1	11.07	6.48	2.00	0.78	0.80	0.83	0.04
MAD2	8.53	5.56	1.81	0.81	0.78	0.81	−0.02
MAD3	6.53	5.02	1.63	0.76	0.74	0.80	0.00
GAC1	9.47	6.00	1.85	0.71	0.76	0.79	0.13
CHD1	10.73	6.23	1.98	0.77	0.80	0.82	0.04
CHD2	10.40	6.16	1.97	0.78	0.80	0.83	0.04
CHD3	11.60	7.15	2.09	0.84	0.83	0.85	−0.01
Average	10.03	6.60	1.98	0.80	0.81	0.84	0.01

*Note:* Genetic diversity parameters in this table represent average values across all SSR loci for each population.

### 4.2 Genetic Distance Among *B. kashmirensis* Populations

**FIGURE 2:**
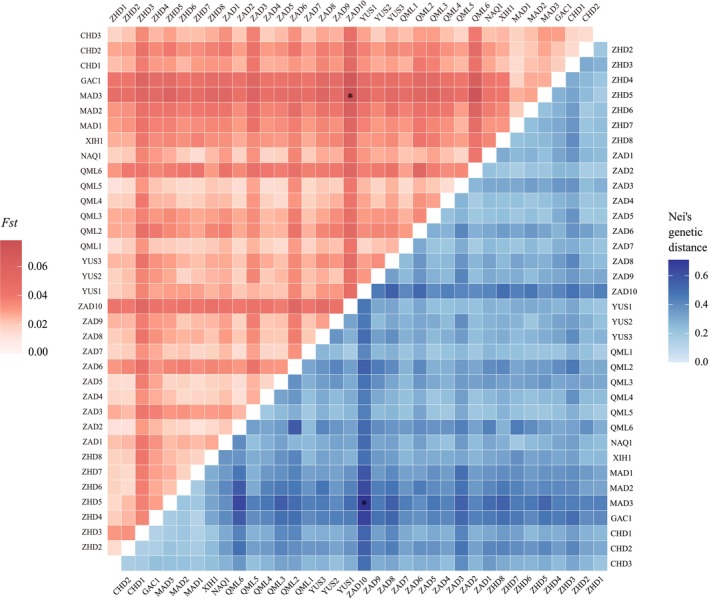
Genetic differentiation among 36 *B. kashmirensis* populations. Pairwise *F*st values are shown above the diagonal, and Nei's genetic distances below. Color intensity reflects the magnitude of differentiation, and asterisks (*) indicate the highest values.

The results of *F*st and Nei's genetic distance analyses among the 36 geographic populations of *B. kashmirensis* showed that *F*st values ranged from 0.013 to 0.078, and Nei's genetic distances ranged from 0.130 to 0.710 (Figure 2, Tables S1 and S2). Some population pairs exhibited relatively large genetic distances, reflecting notable differences in allele frequencies among populations. Notably, the highest Nei's genetic distance and *F*st value were observed between populations MAD3 (located in the eastern part of the study area) and ZAD10 (located in the southwestern part), indicating the greatest genetic distance between these two populations.

### 4.3 Genetic Structure of *B. kashmirensis* Populations

**FIGURE 3:**
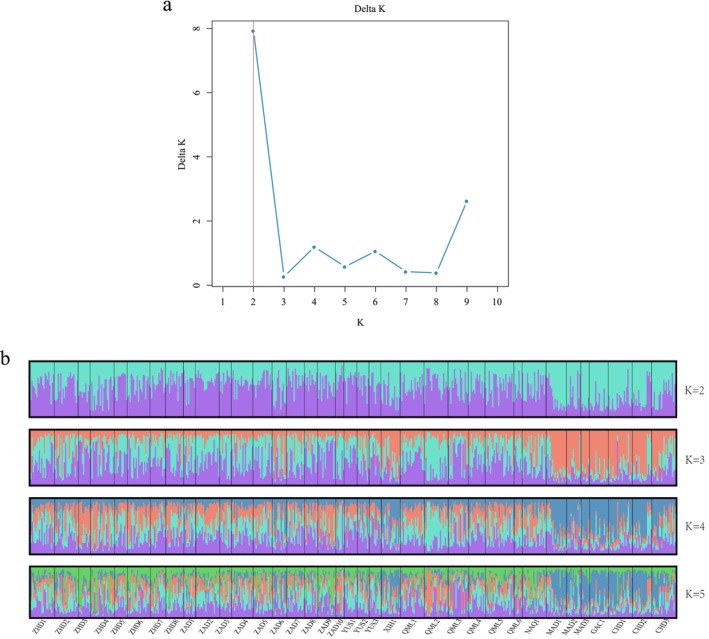
STRUCTURE‐based clustering analysis of *B. kashmirensis* populations. (a) Δ*K* values inferred from STRUCTURE analysis for *K* = 1 to 10, showing the optimal number of genetic clusters; (b) population genetic structure of the 36 populations under *K* = 2 to *K* = 5, illustrating the assignment of individuals to the corresponding clusters (indicated by color).

Bayesian clustering analysis of 36 geographic populations of *B. kashmirensis* was performed using STRUCTURE software, with the number of assumed clusters (*K*) set from 1 to 10. The Δ*K* method identified *K* = 2 as the optimal number of clusters (Figure 3a), indicating that the populations can be divided into two major genetic groups. Clustering patterns for *K* = 2, 3, 4, and 5 were further examined and visualized (Figure 3b). Populations in the eastern part of the study area—including Xinghai County (XIH1), Maduo County (MAD1, MAD2, MAD3), Gangcha County (GAC1), and Chindu County (CHD1, CHD2, CHD3)—primarily belonged to one genetic subgroup, whereas populations from the western region were mostly assigned to the other.

### 4.4 UPGMA Clustering and PCoA Analysis of *B. kashmirensis* Populations

**FIGURE 4:**
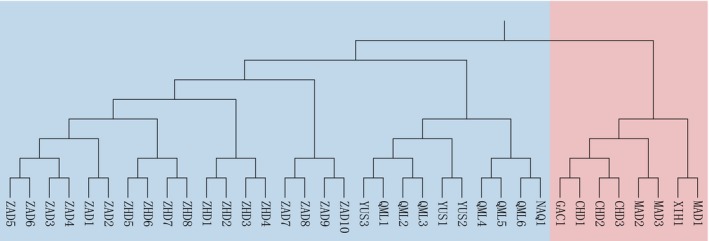
UPGMA clustering of *B. kashmirensis* populations based on Nei's genetic distance. Red and blue shading represent genetic cluster 1 and genetic cluster 2, respectively, reflecting geographic divisions that are consistent with the STRUCTURE and PCoA analyses.

**FIGURE 5:**
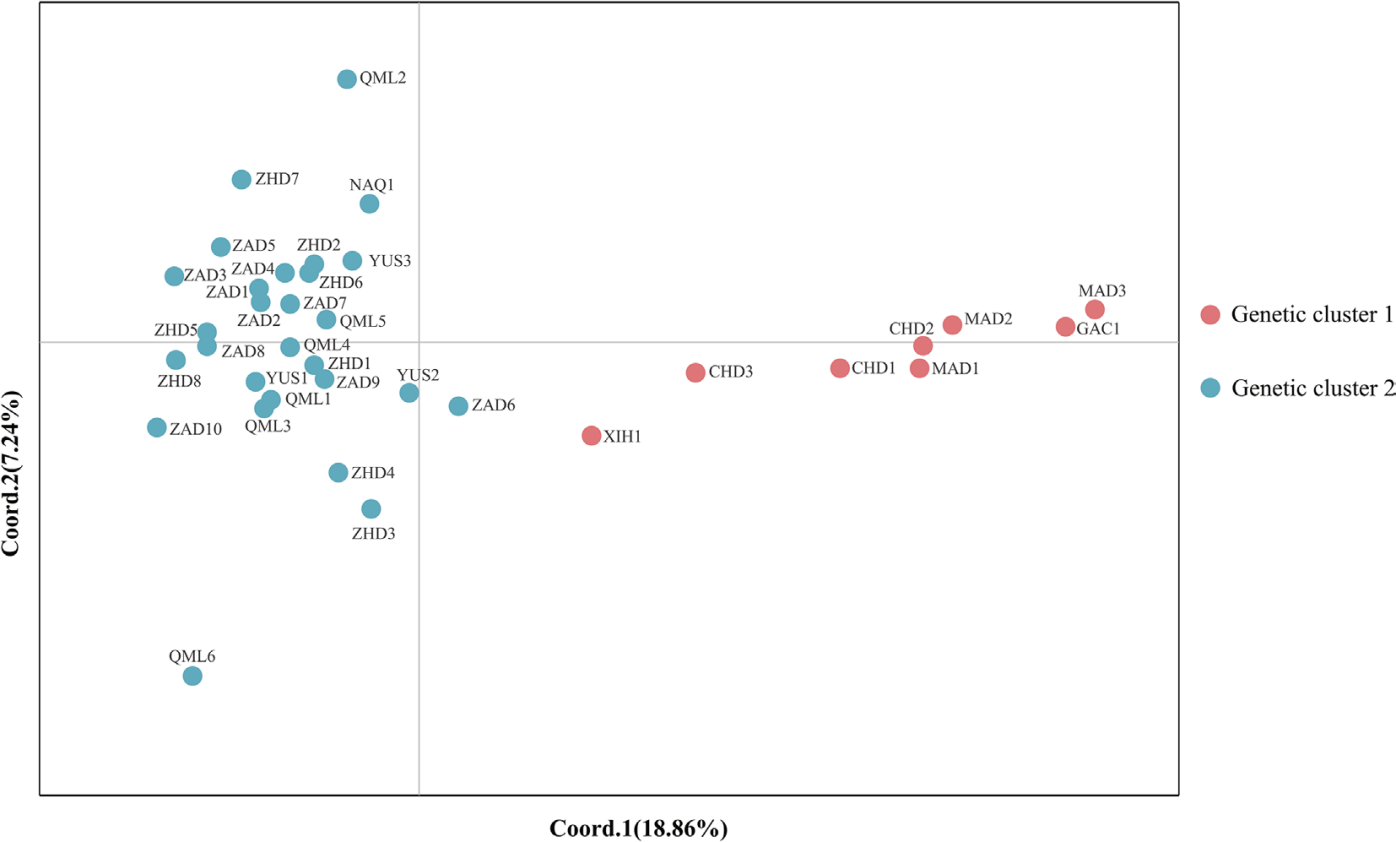
PCoA of *B. kashmirensis* populations based on Nei's genetic distance. Points are colored by genetic cluster.

The results of UPGMA clustering based on Nei's genetic distance (Figure 4) and PCoA (Figure 5) revealed that populations from Xinghai County, Maduo County, Gangcha County, and Chindu County clustered together and were clearly separated from other populations along the coordinate axes. Notably, the spatial configuration of populations in the PCoA plot closely mirrored their geographic distribution, with genetic cluster 1 concentrated in the eastern part of the study area and cluster 2 predominantly located in the western part (Figure 1). These findings are highly consistent with the STRUCTURE analysis results, further confirming the existence of geographically associated genetic differentiation within *B. kashmirensis* populations in this region.

### 4.5 Analysis of Molecular Variance in *B. kashmirensis* Populations

AMOVA was performed based on the population genetic structure inferred at *K* = 2 from STRUCTURE analysis. The results revealed a highly significant genetic structure in *B. kashmirensis* populations (*p* < 0.00001) (Table 4). Of the total genetic variation, 92.32% was attributed to within‐individual variation, indicating high overall genetic diversity within populations. The remaining 7.68% of genetic variation was partitioned among groups (2.00%), among populations within groups (1.42%), and among individuals within populations (4.26%). Although the proportion of variation among groups was relatively small, it exceeded that among populations within groups, indicating low but significant genetic differentiation between eastern and western populations of *B. kashmirensis* .

**TABLE 4:** Analysis of molecular variance of *B. kashmirensis* populations at *K* = 2.

Source of variation	df	Sum of squares	Variance components	Percentage of variation	Fixation indices	*p*
Among groups	2	59.40	0.13031 Va	2.00	FCT = 0.04411	< 0.00001
Among populations within groups	34	317.78	0.09276 Vb	1.42	FSC = 0.01450	< 0.00001
Among individuals within populations	503	3311.83	0.27816 Vc	4.26	FIS = 0.01996	< 0.00001
Within individuals	539	3249	6.02783 Vd	92.32	FIT = 0.07677	< 0.00001

### 4.6 Correlation Between Genetic Diversity and Environmental Factors in *B. kashmirensis* Populations

**FIGURE 6:**
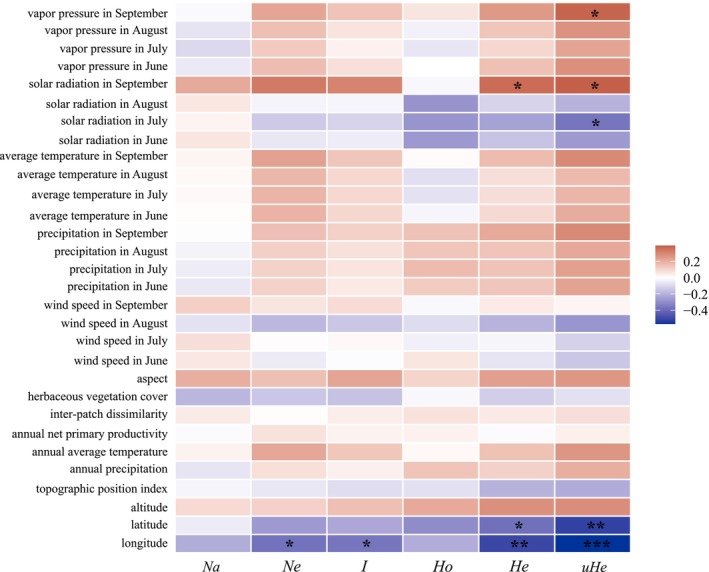
Correlations between population genetic diversity and environmental factors. *N* *a*: number of alleles; *N* *e*: effective number of alleles; *I*: Shannon's information index; *H* *o*: observed heterozygosity; *H* *e*: expected heterozygosity; *uHe*: unbiased expected heterozygosity. *p < 0.05; **p < 0.01; ***p < 0.001.

Pearson correlation analysis (Figure 6) revealed no significant relationships between genetic diversity parameters and elevation, topographic variables, annual NPP, wind speed, precipitation, or temperature. The *N* *e* and *I* were significantly negatively correlated with longitude. The *H* *e* exhibited a significant positive correlation with solar radiation in September, a significant negative correlation with latitude, and a highly significant negative correlation with longitude. The *uHe* was significantly positively correlated with vapor pressure and solar radiation in September, significantly negatively correlated with solar radiation in July, and exhibited highly significant negative correlations with both latitude and longitude. These results suggest that geographic factors (latitude and longitude) are significantly associated with genetic diversity, while climatic factors such as solar radiation and vapor pressure also show correlations with genetic variation. These associations suggest that environmental and climatic conditions may play potential roles in shaping population genetic processes, although this inference requires further investigation.

## 5 Discussion

### 5.1 Genetic Diversity and Environmental Determinants in *B. kashmirensis* Populations

In population genetics research, parameters such as the *N* *a*, *H* *o*, and *H* *e* are fundamental indicators for evaluating genetic diversity and the adaptive potential of populations (Kanaka et al. 2023). In this study, we employed 15 SSR markers to assess the genetic diversity of 539 *B. kashmirensis* individuals from 36 geographic populations across Qinghai Province. The number of alleles per locus ranged from 14 to 42, indicating substantial allelic richness across multiple loci. The *Na* values observed in this study were notably higher than those reported by Meydan et al. (2016) for *B. terrestris* populations in Turkey using five SSR markers. This discrepancy may be attributed to differences in the number of markers used, species‐specific genetic backgrounds, or sample sizes. All SSR loci showed a *PIC* greater than 0.5, with an average value of 0.851, indicating that the selected markers were highly polymorphic and well‐suited for assessing population genetic diversity and structure. Across the 36 populations, the average *I* was 1.98, while the mean values of *H* *o*, *H* *e*, and *uHe* were 0.80, 0.81, and 0.84, respectively, reflecting a relatively high level of genetic diversity in *B. kashmirensis* . Compared to the findings of An (2012), who analyzed 16 populations of *B. pyrosoma* from the eastern Tibetan Plateau and Qinling Mountains, the *B. kashmirensis* populations examined in this study exhibited higher genetic diversity levels. This difference may be attributed to the unique climatic conditions and diverse vegetation types in Qinghai Province, which likely enhance habitat heterogeneity and promote greater evolutionary potential of *B. kashmirensis* (Jackson et al. 2018).

Genetic diversity levels in populations are often correlated with environmental variables such as longitude, latitude, elevation (Lozier et al. 2011), temperature (Martinet et al. 2021), and precipitation (Kardum Hjort et al. 2024). Among these, climatic factors—particularly temperature and precipitation—are generally considered to have a stronger influence on biological evolution (Jin and Liu 2008; Huang et al. 2007). Pearson correlation analysis was used in this study to evaluate the relationships between geographic and environmental variables and the genetic diversity of *B. kashmirensis* populations. Significant or highly significant correlations were detected between genetic diversity indices and environmental variables, including longitude, latitude, solar radiation in July and September, and vapor pressure in September. Specifically, *N* *e*, *I*, *H* *e*, and *uHe* were significantly negatively correlated with longitude, while *H* *e* and *uHe* were also significantly negatively correlated with latitude. This may be due to the lower elevation typically associated with higher longitude and latitude within the study area. These results suggest that higher‐elevation habitats are more favorable for *B. kashmirensis* . Based on previous analysis of suitable habitat assessments for different indicator species bumblebees on the Qinghai–Tibet Plateau under current and future climate scenarios using the MaxEnt model, the results show that future climate warming will shift the suitable range of *B. kashmirensis* toward higher altitudes. Since the Sanjiangyuan region in the southwestern part of our study area is located at a much higher elevation than the eastern region, it may provide more favorable conditions for the survival of *B. kashmirensis* (Liang et al. 2023). In addition, *uHe* was significantly negatively correlated with solar radiation in July, while *H* *e* and *uHe* were positively correlated with solar radiation in September. This likely reflects the influence of intense solar radiation on bumblebee flight behavior in high‐altitude regions. High‐altitude regions in Qinghai are characterized by a generally cold climate, with temperatures in September substantially lower than in July. The minimum ambient temperature suitable for bumblebee activity is approximately 12°C (Heinrich 1972). In September, the maximum temperatures at most sampling sites were significantly below this threshold, requiring bumblebees to rely on solar radiation to acquire heat for maintaining body temperature and sustaining normal flight activity. In contrast, temperatures in July were relatively higher, and excessive solar radiation may cause overheating, thereby disrupting normal flight and foraging behavior, which could subsequently affect their genetic diversity (Hirota and Obara 2000; Bergman et al. 1996). Furthermore, *uHe* was significantly positively correlated with vapor pressure in September. As an indicator of atmospheric humidity, vapor pressure can affect bumblebee flight activity and foraging efficiency (Reeves et al. 2024). Given Qinghai's arid conditions, adequate humidity levels may facilitate bumblebee activity, enhance interactions among individuals, and promote gene flow, thereby contributing to increased population genetic diversity. Since *uHe* corrects for sampling bias in small populations, it provides a more accurate and stable estimate of genetic diversity compared with *H* *e* (Nei 1978). Therefore, the results reflected by *uHe* offer greater stability and reliability when assessing the impact of environmental factors on population genetic diversity.

### 5.2 Genetic Differentiation and Population Structure of *B. kashmirensis*

*F*st is a key metric for quantifying genetic differentiation among populations (Subramanian 2022). According to Wright's criteria (Wright 1965), an *F*st value below 0.05 indicates little genetic differentiation, while values between 0.05 and 0.15 suggest moderate differentiation. In this study, pairwise *F*st values among geographic populations ranged from 0.013 to 0.078, with a mean of 0.032. Specifically, *F*st values for western populations ranged from 0.013 to 0.058 (mean = 0.029), and for eastern populations from 0.016 to 0.042 (mean = 0.025). These results indicate generally low but variable allele frequency differences among populations. Notably, allele frequency differences between eastern and western populations were generally higher than within regions, implying a weak but detectable geographic genetic structure across the species' distribution range. Nei's genetic distance, ranging from 0 to 1, measures genetic similarity, where 0 indicates identical genetic composition and values closer to 1 represent more distant relationships. In this study, Nei's genetic distances among populations ranged from 0.130 to 0.710, suggesting substantial variation in genetic relationships across geographic populations. Notably, populations MAD3 (located in the eastern part of the study area) and ZAD10 (located in the southwestern part) exhibited the highest Nei genetic distance and *F*st values, suggesting the greatest genetic distance between them. This was further supported by PCoA, where MAD3 and ZAD10 were positioned farthest apart along the principal coordinate axes, reinforcing the substantial genetic difference between these two populations. Comprehensive analyses integrating STRUCTURE population genetic structure, UPGMA clustering, and PCoA revealed that the 36 geographic populations of *B. kashmirensis* cluster into two distinct genetic clusters. The spatial configuration of populations in the PCoA plot closely corresponded to their geographic distribution, further supporting the conclusions of this study regarding the eastern and western genetic clusters. Populations in the eastern part of the study area—including Xinghai County (XIH1), Maduo County (MAD1, MAD2, MAD3), Gangcha County (GAC1), and Chindu County (CHD1, CHD2, CHD3)—primarily belong to one genetic cluster, whereas the western populations form the other. Notably, although the three CHD populations are geographically closer to the western region, they were consistently assigned to the eastern genetic cluster in both STRUCTURE and UPGMA analyses. Our allele frequency comparison further supports this pattern: only three alleles showed significant differences between CHD and the eastern populations, whereas 21 alleles differed significantly between CHD and the western populations. This indicates that CHD shares highly similar allele frequency patterns with the eastern group across most loci, while being distinct from the western group at multiple loci, explaining its clustering with the eastern lineage. Although geographically proximate to the western populations, CHD retains more eastern genetic signatures, which may reflect historical gene flow, local adaptation, or the influence of the complex topography of the Qinghai–Tibetan Plateau on gene flow direction and intensity. The specific reasons underlying this result remain unclear and require further investigation to be elucidated. AMOVA based on *K* = 2 grouping showed that, although the proportion of variation among groups was relatively small, it exceeded the variation among populations within groups and was highly significant (*p* < 0.00001). These results further support the existence of significant genetic structure among *B. kashmirensis* geographic populations. Despite evident genetic structuring, both genetic clusters exhibited some degree of admixture, suggesting frequent gene flow among populations throughout their evolutionary history. This pattern is consistent with genetic structure studies of many winged insects (Hu et al. 2023; Takeuchi et al. 2018; Zheng et al. 2013; Fang et al. 2022), where flight capability facilitates dispersal over large spatial scales and promotes gene flow.

## 6 Conclusions

In this study, SSR markers were employed to assess the genetic diversity and population structure of 539 *B. kashmirensis* individuals from 36 geographic populations. The 15 selected SSR loci showed high polymorphism and effectively characterized the genetic variation and structure of *B. kashmirensis* populations. Overall genetic diversity was relatively high, influenced primarily by environmental factors including longitude, latitude, solar radiation in July and September, and vapor pressure in September. Population structure analyses revealed clear genetic differentiation between eastern and western groups, with significant but low‐level genetic structuring.

## Author Contributions

**Rui Zhang:** data curation (lead), formal analysis (equal), investigation (lead), methodology (equal), writing – original draft (lead). **Guo Sun:** data curation (equal), formal analysis (equal), methodology (lead), writing – original draft (equal). **Chengbo Liang:** resources (equal). **Daoxin Liu:** funding acquisition (equal), resources (equal), supervision (equal), writing – review and editing (equal). **Jingyan Yan:** formal analysis (equal), funding acquisition (lead), resources (equal), supervision (equal), writing – review and editing (equal).

## Funding

This research was supported by the Natural Science Foundation of Qinghai Science and Technology Department (2024‐ZJ‐925) and the Open Project of the Qinghai Provincial Key Laboratory of Animal Ecological Genomics (QHEG‐2025‐06).

## Conflicts of Interest

The authors declare no conflicts of interest.

## Supporting information


**Table S1:** ece372876‐sup‐0001‐TableS1.xlsx.

**Table S2:** ece372876‐sup‐0002‐TableS2.xlsx.

## Data Availability

The microsatellite (SSR) genotyping dataset for *Bombus kashmirensis* used in this study has been deposited in the Dryad Digital Repository and is available at https://doi.org/10.5061/dryad.jdfn2z3qj.
